# Nursing Care in Peripheral Intravenous Catheter (PIVC): Protocol of a Best Practice Implementation Project

**DOI:** 10.3390/nursrep12030049

**Published:** 2022-07-13

**Authors:** Fernando Catarino, Cristina Lourenço, Célia Correia, João Dória, Maria Dixe, Cátia Santos, Joana Sousa, Susana Mendonça, Daniela Cardoso, Cristina R. Costeira

**Affiliations:** 1 Cova da Beira University Hospital Center, Alameda Pêro da Covilhã, 6200-251 Covilhã, Portugal; fernando.catarino@gmail.com (F.C.); comclourenco@chcbeira.min-saude.pt (C.L.); celiapaulo29@gmail.com (C.C.); joaodoria1977@gmail.com (J.D.); 2 Centre for Innovative Care and Health Technology (ciTechCare), Rua de Santo André—66–68, Campus 5, Polytechnic of Leiria, 2410-541 Leiria, Portugal; maria.dixe@ipleiria.pt (M.D.); catia.santos@ipleiria.pt (C.S.); joana.sousa@ipleiria.pt (J.S.); susana.mendonca@ipleiria.pt (S.M.); 3 School of Health Sciences of Polytechnic of Leiria, Campus 2, Morro do Lena, Alto do Vieiro, Apartado 4137, 2411-901 Leiria, Portugal; 4 Portugal Centre for Evidence-Based Practice: A JBI Centre of Excellence, 3000 Coimbra, Portugal; dcardoso@esenfc.pt; 5 The Health Sciences Research Unit: Nursing (UICISA:E), Nursing School of Coimbra (ESEnfC), 3004-011 Coimbra, Portugal

**Keywords:** peripheral venous catheter, best practices, implementation science, nursing care, care quality, patient safety

## Abstract

Background: The use of a peripheral intravenous catheters (PIVC) is a common invasive practice in healthcare settings. It is estimated that about 70% of people with PIVCs will develop associated complications, such as infections. It is the consensus that best practices could reduce the appearance of such complications and reduce the length of stay in hospital. Methods: A project will be applied to implement the best approach in peripheral venous catheterization, provided by clinical nurses from an inland hospital in Portugal. The Joanna Briggs Institute methodology will be used on evidence implementation projects, which will be developed in three phases. First, a baseline audit will be performed. The second phase implements corrective measures, and the third phase is a follow-up audit. Conclusions: This project will improve the practice of the nursing team on peripheral venous catheterization nursing cares, positively influencing the quality of nursing care and patient safety. The implementation and dissemination of this project could boost its replication in other centres.

## 1. Introduction

The use of a peripheral intravenous catheter (PIVC) is a common invasive practice in health contexts, intending to favour the person’s treatment and recovery more quickly and promote their comfort due to the need for frequent intravenous medication administration; however, it is prone to complications [1,2,3,4]. In 2020, a systematic review and meta-analysis study was conducted that indicated the most common complications related to PIVC were phlebitis (with definition) 19.3%, phlebitis (without definition) 4.5%, infiltration/extravasation 13.7%, occlusion 8%, leakage 7.3%, pain 6.4% and dislodgement 6.0% [5].

The complications related to PIVC are more embracing than those mentioned above; it is estimated that about 70% to 90% of people with PIVC will develop associated complications [6,7], which may increase the length of stay in a hospital ward by about 22 days [8,9].

To reduce complications, it is the consensus and a priority to verify compliance with the evidence-based recommendations aimed at the insertion, removal and maintenance of these devices [10,11,12]. This protocol aims to disclose the methodology that will be applied in the evidence implementation project that can be applied in different clinical contexts in which PIVCs are used.

The implementation project aims to promote evidence-based practices regarding nursing care in peripheral intravenous catheterization.

The specific objectives will be:

- To assess current compliance with evidence-based criteria of nursing care in the insertion, fixation, maintenance and surveillance of peripheral intravenous catheters;
- To identify barriers and enablers to achieving compliance;
- To develop strategies to deal with areas of non-compliance;
- To improve knowledge about best practices related to peripheral intravenous catheter care;
- To improve compliance with evidence-based criteria for peripheral intravenous catheter care;
- To improve outcomes concerning peripheral intravenous catheter care.

## 2. Materials and Methods

The methodology described by Joanna Briggs Institute Practical (J.B.I.) in implementing evidence projects [13] consists of performing at least two audits for the data collection process. The audit process developed in clinical settings is extensively used as an effective strategy to identify errors, monitor study operations and ensure high-quality data [14].

In this evidence implementation project, the J.B.I. framework will be used for promoting evidence-based healthcare that involves three phases of activity [13]:

(i) Establishing a team for the project and undertaking a baseline audit based on criteria informed by the evidence;
(ii) Reflecting on the baseline audit results and designing and implementing strategies to address non-compliance found in the baseline audit;
(iii) Conducting a follow-up audit to assess the outcomes of the interventions implemented to improve practice and identify future practice issues to be addressed in subsequent audits.

### 2.1. Phase 1: Stakeholder Engagement (or Team Establishment) and Baseline Audit

Phase 1 aims to establish the team for the project and undertake a baseline audit based on criteria informed by the evidence [13]. This phase of the evidence implementation project will be conducted in three months.

The team implementation project will include two coordinators, one experiment J.B.I. research supervisor and four registered nurses (one of them is the Chief Nurse of the setting of interest); their positions, organizations and roles will be presented in Table 1.

Table 2 presents the audit checklist based on evidence criteria provided by J.B.I., which will be used at baseline and follow-up audits. The requirements provided are based on one J.B.I. Evidence Summaries: “peripheral intravenous catheter (pivc): general care and catheter lumen patency” [7]; “peripheral intravenous catheter (pivc) care: dressings and catheter securement” [12]; “peripheral intravenous catheter (pivc) care: insertion” [10]; “peripheral intravenous catheter (pivc) care: removal and replacement” [11].

An “audit plan” was developed and given to all audit nurses for orientation, including audit date, auditor identification, audit time, references for the audit, and the criteria evaluation method. The criteria mensurable will be defined using observation of nursing technique (*) and clinical records through *SClínico*^®^ (Portugal software used to clinical reports) (**).

The checklist allows the response “yes” when it is verified in the criteria, “no” when is not verified the in criteria or “not applicable (n/a)” when it does not apply to the audit criteria (e.g., on PIVC insertion the removal criteria will not applied).

The audit process will be developed until a minimum of a sample size of *n* = 20 in all audit areas. The sample size was calculated taking into account the number of beds in the service and all patients having at least one PIVC in order to respect the recommended times of three months for phase 1.

### 2.2. Phase 2: Design and Implementation of Strategies to Improve Practice

In the second phase, all results from the baseline audit will be used to create an audit report. Feedback will be given to stakeholders and the nursing team from the setting through email and an online meeting (*Microsoft Teams*^®^).

To understand the barriers that lead to the gap between current practice and the best practice found by the baseline audit, all teams will design strategies to promote the best practices. This phase of the evidence implementation project will be conducted for three months.

### 2.3. Phase 3: Follow-Up Audit Post-Implementation of Change Strategy

In the last phase, a follow-up audit will be conducted that aims to measure if any improvement in compliance with best practice has been reached and recognize any areas that need additional focus and progress based on the framework of the Deming cycle (PDCA—Plan–Do–Check–Act) [15].

The follow-up audit will be performed using the same audit checklist used on the baseline audit. The baseline audit data will be compared with follow-up audit data to analyse any change in compliance rates. This phase of the evidence implementation project will be conducted for three months.

## 3. Conclusions

The importance of respecting good nursing practices in the care of people with PIVC are indicators of the quality of care and guarantees their safety. The implementation and dissemination of this project could boost its replication in other centres and even extend to all clinical units where PIVC is a procedure used, contributing to reducing Infections Associated with Healthcare (HAIs).

## Figures and Tables

**Table 1:** Team members, their positions, organizations and roles in the implementation project.

Team Member	Position	Organization	Role
Coordinators: Nurse 1 * Nurse 2 **	Registered nurses and Researchers	* Cova da Beira University Hospital Center ** School of Health Sciences of Polytechnic of Leiria	Project coordinator Outlined and monitored clinical Audit project Data analysis and Report Training the stakeholders
Collaborator Nurse 4	J.B.I. Researcher	The Health Sciences Research Unit: Nursing	Supervision Technical support
Collaborator Nurse 5	Chief Nurse	Study Setting	Clinical facilitators (champions)
Stakeholders Nurse 5 Nurse 6 Nurse 7	Registered nurse	Study Setting	Clinical facilitators (champions) Training List barriers Designed strategies Develop some strategies

**Table 2:** Audit checklist with best practice criteria.

PERIPHERAL VENOUS CATHETER (PIVC) CARE AUDIT CHECK-LIST				
Audit Areas	Audit Criteria	Yes	No	n/a
PIVC insertion	Is the aseptic technique used in PIVC insertion? *			
	Is the puncture site cleaned with antiseptic? *			
	In patients with low pain threshold, is the use of a topical anaesthetic considered prior to the puncture? *			
Dressings and catheter securement	Is transparent film used at the catheter insertion site? *			
	Is a sterile compress used if blood or exudate? *			
	Is the dressing renewed whenever it is wet, dirty or peeling off? *			
Removal and Replacement	Do you remove the catheter because it has no clinical indication, does it malfunction or shows signs of phlebitis? *			
	After removal does it exert firm pressure on the site? *			
	The integrity of the peripheral venous catheter is verified after its removal *			
	Did you record the catheter removal? **			
	Did you mention the reason for removal, length of stay and site assessment in the register? **			
General care and catheter lumen patency	Is the PIVC site inspected every 4 h in hospitalized adult patients? *			
	Is the PIVC site inspected in critically ill patients inspected every 2 h? *			
	Is the visual of the PIVC by contact with local (palpation)? *			
	Hand hygiene before and after contact with the PIVC? *			
	Aseptic technique is used during catheter care *			
	Before administration of therapy, is aspiration performed to check for PIVC patency? *			
	After each use PIVCs are flushed and locked, or at minimum of once daily if not in use. *			
	Is the unused catheter flushed at least once per shift? *			
	Is saline solution used in catheter washing? *			
	Is the amount of solution at least twice the internal volume of the catheter? (e.g., minimum 5ml) *			
	The Institution has a protocol for the management of PIVC **			

* Corresponde to observation of nursing technique; ** Corresponde to consultation of clinical records through SClínico^®^.

## Data Availability

Not applicable.
